# Nanoformulation of the K-Ras(G12D)-inhibitory peptide KS-58 suppresses colorectal and pancreatic cancer-derived tumors

**DOI:** 10.1038/s41598-023-27825-8

**Published:** 2023-01-10

**Authors:** Kotaro Sakamoto, Yun Qi, Eijiro Miyako

**Affiliations:** 1 Research & Development Department, Ichimaru Pharcos Company Limited, 318-1 Asagi, Motosu, Gifu 501-0475 Japan; 2 Graduate School of Advanced Science and Technology, Japan Advanced Institute of Science and Technology, 1-1 Asahidai, Nomi, Ishikawa 923-1292 Japan

**Keywords:** Nanomedicine, Cancer, Drug delivery, Pharmaceutics

## Abstract

Single amino acid mutations of Ras occur in 30% of human cancers. In particular, K-Ras(G12D) has been detected in the majority of intractable colorectal and pancreatic cancers. Although efforts to target K-Ras(G12D) are currently underway, no effective drugs are available. We previously found that the K-Ras(G12D)-inhibitory bicyclic peptide KS-58 exhibits antitumor activity against syngeneic colon and orthotopic grafted pancreatic tumors; however, pristine KS-58 is difficult to handle because of low water solubility and it requires frequent administration to obtain sufficient antitumor activity. In this study, we used a nanoformulation of KS-58 prepared with the highly biocompatible surfactant Cremophor^®^ EL (CrEL) to improve water solubility and reduce the dosing frequency. Nanoformulations of KS-58 with CrEL dramatically improved its water solubility and increased its stability. Weekly intravenous administration of KS-58 nanoparticles (NPs) suppressed the growth of CT26 and PANC-1 cell-derived tumors in vivo, and fluorescent bioimaging indicated that the NP-encapsulated near-infrared fluorescent probe indocyanine green selectively accumulated in the tumor and was safely excreted through the kidneys following intravenous injection. Histopathological analysis of CT26 tumors and Western blotting of PANC-1 tumors revealed that KS-58 NPs reduced ERK phosphorylation, a downstream signal of K-Ras(G12D). Our results suggest that KS-58 NPs represent a novel therapeutic agent for treating colorectal and pancreatic cancers.

## Introduction

Since the discovery of single amino acid mutations of Ras in human cancers, 30 years of research has been focused on developing strategies to ameliorate the effects of mutated Ras^1,2^. In 2021, a covalent small molecule drug was identified that targeted K-Ras(G12C), a mutated form of Ras primarily found in lung cancer^3^. A number of preclinical and human clinical studies are currently underway to evaluate the anti-cancer activity of K-Ras(G12C) inhibitor in combination with other chemotherapeutic drugs^4–6^; however, the development of drugs against K-Ras(G12D) in colorectal and pancreatic cancers, which are known as intractable cancers, have not been as successful^7^. A major reason is that covalent small molecule drug design is difficult to apply to K-Ras(G12D)^8^. By structurally modifying the K-Ras(G12C) inhibitor, a non-covalent K-Ras(G12D) inhibitor MRTX1133 was developed in late 2021, but twice-daily intraperitoneal administration was required to yield anti-cancer activity in vivo^9^.

We attempted to synthesize a K-Ras(G12D) inhibitor using cyclic peptides. In 2017, the first K-Ras(G12D)-selective inhibitory peptide “KRpep-2d” was reported^10–12^. We also identified a K-Ras(G12D)-inhibitory bicyclic peptide “KS-58”, which is a derivative of KRpep-2d, and reported its in vitro and in vivo growth inhibition activities against CT26 (mouse colorectal cancer cell line) and PANC-1 (human pancreatic cancer cell line) cells both expressing K-Ras(G12D)^13,14^. However, there are several challenges associated with the development of KS-58. One is its poor water solubility and the other is the need for frequent dosing. With respect to the latter, the in vivo antitumor activity of KS-58 requires intravenous administration once every 2 days at a dose of 40 mg/kg because of its poor pharmacokinetics. The elimination half-life (t_1/2_) of KS-58 in the blood is approximately 30 min^14^. Therefore, it is necessary to reduce the frequency of administration from once every 2 days to once a week to reduce the burden on patients.

Several strategies using chemical conjugations with albumin-binding tags^15,16^ or polyethylene glycol^17,18^ have been shown to improve the pharmacokinetics of various peptides. Such conjugations can reduce excretion from the kidneys and prolong t_1/2_ in the blood; however, these strategies have been applied mostly to peptides with extracellular targets. Moreover, peptide drug conjugations exhibit reduced intracellular penetration with increasing molecular weight. Therefore, we evaluated nanoformulations to further improve the solubility, t_1/2_ in the blood, and the intracellular penetration of the peptide drugs^19^. Nanoparticles (NPs) in the range of 10–100 nm in diameter have been considered effective drug carriers because they are able to accumulate in solid cancer tissues owing to the enhanced permeability and retention (EPR) effect^20^.

**Figure 1:**
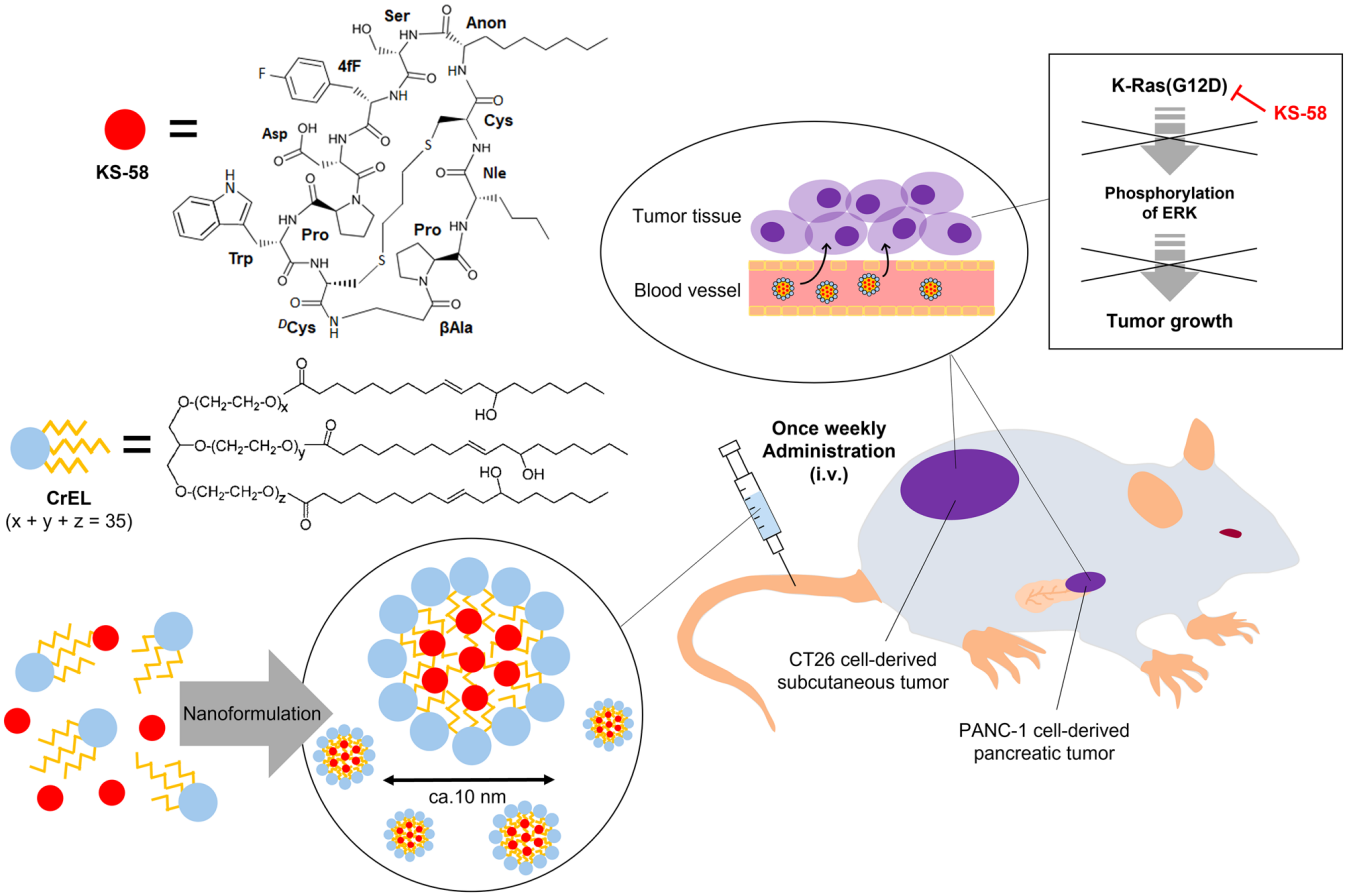
Schematic diagram for this study.

In this study, we evaluated a nanoformulation of KS-58 with Cremophor^®^ EL (CrEL), a biocompatible surfactant approved as a pharmaceutical additive^21^. KS-58 was stably encapsulated into CrEL NPs. The resulting KS-58 NPs exhibited significant antitumor activity against CT26 colorectal cancer cell-derived syngeneic tumors and PANC-1 pancreatic cancer cell-derived orthotopic tumors following weekly intravenous (i.v.) administration (Fig. 1). The design and concept of KS-58 nanoformulations represent a significant advancement in the effective targeting of K-Ras(G12D).

## Materials and methods

### Preparation of KS-58 NPs

A K-Ras(G12D)-inhibitory peptide KS-58 (1333.6 g/mol) was synthesized by SCRUM, Inc. (Tokyo, Japan) as previously reported^13^. Following reverse phase-high performance liquid chromatography (RP-HPLC) purification, a structural assignment was performed by matrix-assisted laser desorption ionization-time of flight mass spectrometry. Cremophor^®^ EL (CrEL) (09727-14) was purchased from Nacalai Tesque, Inc. (Kyoto, Japan). KS-58 (200 mg) was dissolved in DMSO (2 mL) at a concentration of 100 mg/mL. Then, 10 × D-PBS (048-29805, FUJIFILN Wako Pure Chemical Corporation, Osaka, Japan) (3.5 mL) was mixed with purified water (3.5 mL) for the preparation of a 50% 10 × D-PBS solution in a centrifuge tube (15 mL size). CrEL (1 mL) was added to the 50% 10 × D-PBS solution and mixed well by a vortex mixer. The peptide solution was added dropwise to the CrEL/10 × D-PBS solution, which was being agitated by a vortex mixer at 1,000 rpm. The peptide/CrEL/10 × D-PBS solution was converted to a suspension by repeated vortex agitation and sonication at 50 °C. After repeated chilling on ice, sonication under cold water, and agitation by vortexing, the solution became a transparent KS-58 (20 mg/mL, 15 mM)/DMSO (20%)/CrEL (10%)/10 × D-PBS (35%) solution and was stored at 4 °C until use. DMSO without KS-58 was used for the preparation of control NPs.

### Structural characterization of KS-58 NPs

Negative staining was used to observe the morphology and structure of NPs using high-resolution TEM (H-7600; Hitachi, Tokyo, Japan) at an acceleration voltage of 100 kV. The sample on a TEM grid was tightly washed to remove impurities before the observation. The particle size of the NPs was measured in a disposable cuvette by dynamic light scattering (DLS) with a Zetasizer Nano ZSP (Malvern Instruments Ltd, Malvern, UK).

### In vitro cell growth assay

The CT26.WT (CRL-2638) mouse colorectal cancer and PANC-1 (ATCC CRL-1469) human pancreatic cancer cell lines were purchased from the American Type Cell Collection (Manassas, VA, USA). The cells were cultured in medium supplemented with 10% fetal bovine serum according to the manufacturer’s recommended protocol.

The effect of KS-58 on the proliferation of CT26 and PANC-1 cells was determined following a 72-h exposure to KS-58 with or without nanoformulation. Cells were seeded at 1000 cells/well in 96-well tissue culture plates and allowed to adhere overnight. KS-58 was dissolved in DMSO and diluted with each growth medium with a final DMSO concentration of 0.5%. KS-58 NPs were diluted with each growth media to a final DMSO concentration of 0.04% and a final CrEL concentration of 0.02%. The medium in the wells was removed and replaced with diluted sample solution and the cells were incubated for 3 days. During the incubation, medium containing the samples was replaced each day. The relative cell numbers were estimated using the CellTiter-Glo assay kit (G7570, Promega, WI, USA) according to the manufacturer’s instructions. Luminescence was detected using a SpectraMax i3x (Molecular Devices, CA, USA). The cell proliferation rate (%) was calculated based on cell numbers at day 0 as 0% and at day 3 and treatment with DMSO (a final concentration of 0.5%) or control NPs (a final DMSO concentration of 0.04% and a final CrEL concentration of 0.02%) was designated as 100%.

### In vitro fluorescence microscopy imaging

CT26 cells were seeded in imaging dishes (AGC Techno Glass, Shizuoka, Japan) and allowed to adhere overnight. The attached cells were treated with PBS containing indocyanine green (ICG) and CrEL (ICG, 0.1 mg/mL; and CrEL, 0.5%) for 24 h at 37 °C. After washing with PBS, the cells were observed using a fluorescence microscopy system (IX73) and cellSens V3.1 software (Olympus, Tokyo, Japan) equipped with a mirror unit (IRDYE800-33LP-A-U01; Semrock, Lake Forest, IL, USA) and an objective (40 × magnification, aperture 0.95; UPLSAPO40X, Olympus) at 20 °C.

### In vivo fluorescence bioimaging

To monitor the chronological changes in fluorescence intensity resulting from the tumor-targeting effects of NPs, CT26 tumor-bearing mice (female; 6 weeks; n = 4; average weight = 18 g; average tumor size = 100 mm^3^; BALB/cCrSIc; Japan SLC) were injected i.v. with 250 μL PBS containing ICG and CrEL (ICG, 1 mg/mL; and CrEL, 5%). The mice were euthanized and the major organs, including the heart, liver, spleen, and kidneys, in addition to the tumors, were imaged using an in vivo fluorescence imaging system (VISQU InVivo Smart-LF, Vieworks, Anyang, Republic of Korea) with a 3-s exposure time and an ICG filter (Ex, 740–790 nm; Em, 810–860 nm) at 4, 6, 8, and 12 h post injection. The fluorescence images were acquired and analyzed using CleVue software.

### In vivo antitumor evaluation

Experimental procedures that involved animals and their care were conducted in compliance with the Guide for the Care and Use of Laboratory Animals^22^ and the ARRIVE guidelines^23,24^. The animal experiments using allograft mouse models were conducted following the protocols approved by the Institutional Animal Care and Use Committee of the Japan Advanced Institute of Science and Technology (JAIST) (No. 04-002). Female BALB/cCrSlc mice (4 weeks old; average body weight = 15 g) were obtained from Japan SLC (Hamamatsu, Japan). CT26 cell-derived mouse tumors were generated by injecting 100 μL Matrigel culture medium (v/v = 1:1; Dow Corning, Corning, NY, USA) containing 1 × 10^6^ cells into the dorsal right side of the mice. After approximately 1 week, when the tumor volume had reached ∼ 50 mm^3^, the mice (n = 4 in each group) were intravenously injected with D-PBS (vehicle group), D-PBS containing KS-58 NPs (KS-58 concentration = 20 or 40 mg/kg), or D-PBS containing control NPs once a week. Tumor formation and overall health (viability and body weight) were monitored on the indicated days. Tumor volume was calculated by the formula V = L × W^2^/2, where L and W denote the length and width of the tumor, respectively. When the tumor volume reached > 3000 mm^3^, the mice were euthanized according to the JAIST Institutional Animal Care and Use Committee guidelines. After the mice were euthanized, the liver, kidney, spleen, and CT26 tumors were harvested for immunohistochemical (IHC) staining.

The animal experiments using mouse xenograft models were carried out at UNITECH Co. Ltd. in accordance with the guidelines of the Animal Care and Use Committee of UNITECH (approval No. IMF-220525A-30). To establish orthotropic xenograft tumor models, 2.5 × 10^6^ cells/50 µL of PANC-1 cells were injected into the pancreas of BALB/cAJcl-nu/nu male mice (8 weeks old, CLEA Japan, Inc.). The mice (n = 8 in each group) were intravenously injected with D-PBS containing KS-58 NPs (KS-58 concentration = 20 or 40 mg/kg), or D-PBS containing control NPs (sham and vehicle groups) once a week for 6 weeks. After the mice were euthanized, the pancreas, liver, and kidneys were collected and their weights were measured.

### Immunohistochemical staining of issue sections

IHC analysis of liver, kidney, spleen, and CT26 tumors collected from allograft model mice was performed by the Biopathology Institute Co., Ltd. (Oita, Japan) using standard protocols. Briefly, primary tumors were surgically removed, fixed in 10% formalin, processed for paraffin embedding, and cut into 3–4-μm sections. After incubation with primary antibody, the sections were stained with hematoxylin and eosin and examined by light microscopy (IX73). Anti-digoxigenin-peroxidase (polyclonal from sheep) (S7100; Merck Millipore, Rahway, NJ, USA) and Phospho-p44/42 MAPK (Erk1/2) (Thr202/Tyr204) (D13.14.4E) XP^®^ (monoclonal from rabbit) (4370S; Cell Signaling Technology, Danvers, MA, USA) were evaluated by terminal deoxynucleotidyl transferase dUTP nick end labeling (Tunel) and phospho-p44/42 MAPK antibody, respectively.

### Western blotting of PANC-1 tumors

Western blotting was conducted according to previously reported methods^14^. Experiments were conducted at UNITECH. Briefly, frozen PNAC-1 tumors excised were homogenized in cold RIPA buffer (182-02451, FUJIFILM Wako Pure Chemical Co., Osaka, Japan) supplemented with cOmplete mini EDTA-free Protease Inhibitor Cocktail (11836170001, Roche, Basel, Switzerland) and PhosSTOP using a BioMasher II disposable homogenizer (Nippi, Inc., Tokyo, Japan). The supernatant containing target proteins was collected and total protein concentration was measured. Supernatant proteins (30 μg per gel lane) were separated by SDS-PAGE using Laemmli mini gels (90 mm × 80 mm, 12.5% polyacrylamide gel) and electrophoretically transferred onto polyvinylidene difluoride membranes. Membranes were blocked in TBS containing 0.1% Tween 20 (TBST) plus 3% BSA and then incubated with Phospho-p44/42 MAPK (Erk1/2) (Thr202/Tyr204) (D13.14.4E) XP^®^ in TBST plus 1% BSA. Blotted membranes were then incubated with horseradish peroxidase (HRP)-conjugated mouse anti-rabbit IgG (4090-05, SouthernBiotech, Birmingham, AL, USA) in TBST plus 1% BSA. The reactions were detected with ImmunoStar Zeta (FUJIFILM Wako) and Hyperfilm ECL (Cytiva, Marlborough, MA, USA). After detection, membrane was incubated with stripping buffer (62.5 mM Tris–HCl, 2% SDS, 100 mM 2-mercaptoethanol, pH 6.5) and then washed five times with TBST for measurement of GAPDH as described above using anti-GAPDH antibody (1000-fold dilution) (ab8245, abcam). Protein bands were quantified using ImageJ (NIH, Bethesda, MD, USA). Full length blots are shown in Supplementary Fig. S3.

### Statistical analysis

Multiple group comparisons were made using a one-way or two-way analysis of variance followed by Dunnett's test. All statistical analyses were performed using GraphPad Prism 6 software (GraphPad Software Inc., La Jolla, CA). Values of *p* < 0.05 were considered statistically significant.

## Results and discussion

### Nanoformulation of KS-58

**Figure 2:**
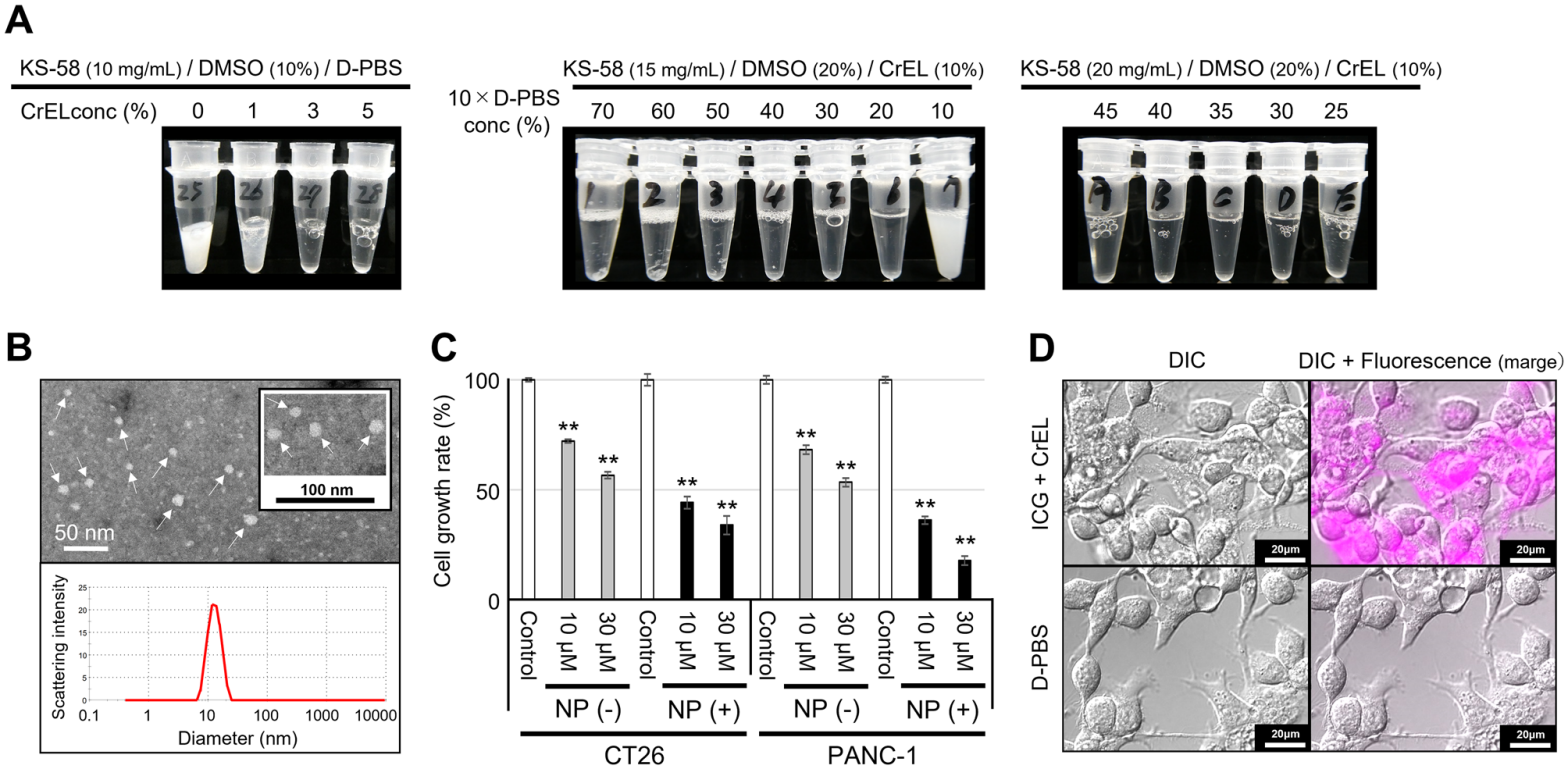
Preparation of a nanoformulation of KS-58 using CrEL and its characterization. (**A**) Examination of the water solubility of KS-58 using the surfactants CrEL and 10 × D-PBS. Appearance of the final formulation; KS-58 (20 mg/mL)/DMSO (20%)/CrEL (10%)/10 × D-PBS (35%) immediately after preparation is shown. (**B**) TEM analysis and size distribution of KS-58 NPs. The hydrodynamic diameter of the KS-58 NPs was approximately 12.95 nm. White arrows represent NPs. (**C**) In vitro growth suppression activity of KS-58 with or without nanoformulation in CT26 and PANC-1 cells (n = 4, ± SEM, **p < 0.01 vs. each control by Dunnett’s test). The relative cell proliferation of each group is shown as % values compared with the control (0.5% DMSO or control NPs), which was set at 100%. (**D**) Cellular uptake of CrEL NPs. ICG was encapsulated in CrEL NPs instead of KS-58 and added to CT26 cells.

As KS-58 is a hydrophobic bicyclic peptide (Fig. 1), we hypothesized that when mixed with biocompatible surfactant, its water solubility would be enhanced by spontaneous invasion within the hydrophobic core of the micelle formed by the surfactant. CrEL (Fig. 1) was selected as the material for KS-58 nanoformulation because it is FDA-approved as a safe pharmaceutical additive. As shown in Fig. 2A, the transparency of the KS-58 (10 mg/mL) solution was increased in a CrEL concentration-dependent manner. KS-58 (10 mg/mL)/DMSO (10%)/CrEL (5%)/D-PBS was found to be prepared as clear aqueous solution (left picture). We wanted to further prepare the solution to a double concentration, KS-58 (20 mg/mL)/DMSO (20%)/CrEL (10%)/D-PBS, but this proved difficult. Because KS-58 (15 mg/mL)/DMSO (20%)/CrEL (10%)/D-PBS (= 10% concentration of 10 × D-PBS) was already cloudy (middle picture). During the process of preparing higher concentrations of KS-58 solution, we found that the composition of D-PBS enhanced KS-58 solubility (Fig. 2A). When KS-58 (15 mg/mL) was dissolved in DMSO (20%)/CrEL (10%)/10 × D-PBS (70–10%), precipitation occurred at 50%–70% concentrations of 10 × D-PBS and white turbidity was observed at 10% concentrations of 10 × D-PBS. In contrast, transparent KS-58 solutions were prepared at 20–40% concentrations of 10 × D-PBS. Higher concentration and transparent KS-58 solutions were prepared at KS-58 (20 mg/mL)/DMSO (20%)/CrEL (10%)/10 × D-PBS (45–25%), and the final concentration of 10 × D-PBS was optimal at 35%. This corresponds to 3.5 times higher salt concentration compared with that of regular D-PBS. However, in actual administration, the final formulation would be diluted (e.g. fivefold), so the salt concentration is not excessive. KS-58 (20 mg/mL)/DMSO (20%)/CrEL (10%)/10 × D-PBS (35%) did not precipitate or cloud for at least 24 h after dilution with 5% glucose, saline, D-PBS, or purified water (Supplementary Fig. S4). Since KS-58 is a medicinal ingredient and there is no need to separate KS-58 incorporated into CrEL micelles from KS-58 that is not incorporated, the method of preparing KS-58 NPs did not include a purification process. Namely, KS-58 NPs solution was used as the total sample with free KS-58 together.

TEM analysis of KS-58/CrEL revealed the formation of unique spherical nanoparticles with a diameter of 10 nm (Fig. 2B). Particle size distribution measurements using DLS indicated that the average hydrodynamic diameter of KS-58 NPs was approximately 12.95 nm (Fig. 2B), which is slightly larger than that of control NPs (diameter = approximately 10.02 nm, Supplementary Fig. S1).

### In vitro anti-cancer efficacy of KS-58 NPs

Cell growth suppression activity of KS-58 with or without nanoformulation was evaluated in CT26 and PANC-1 cells (Fig. 2C). KS-58 NPs or KS-58 alone were added at the same final KS-58 concentration and the number of viable cells was measured after 72 h. Interestingly, the cell growth suppression activity of KS-58 NPs was markedly higher compared with that of KS-58 alone without nanoformulation, presumably because NPs were effectively taken up by the cells (Fig. 2D). To confirm stability of KS-58 encapsulated into CrEL micelles, we evaluated the cell growth suppression activity of KS-58 NPs immediately after preparation, after storage at 4 °C for 1 month, and after storage at 25 °C for 1 month. It was found that their cell growth suppression activities were not significantly different (Supplementary Fig. S1A). This means that KS-58, both incorporated into CrEL micelles and not incorporated into CrEL micelles, is stable for at least 1 month. Additionally, KS-58 NPs solution remained clear 1 month after preparation (Supplementary Fig. S1B). When KS-58 is stably encapsulated, the KS-58 NP solution is clear and does not precipitate or become cloudy. When KS-58 is no longer encapsulated, KS-58 cannot dissolve completely in the aqueous solution, resulting precipitation or cloudiness will be expected. Thus, KS-58 was stably encapsulated within the micelles and retained its cell growth suppression activity.

### Tumor targeting of NPs

**Figure 3:**
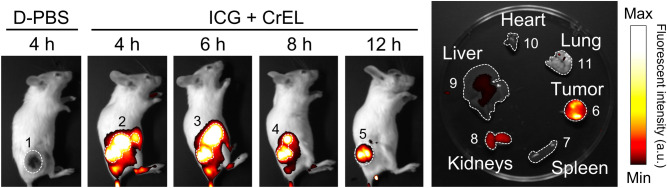
Pharmacokinetic evaluation of CrEL NPs by in vivo bioimaging. A near-infrared fluorescent dye ICG was encapsulated in CrEL NPs instead of KS-58 for pharmacokinetic studies. ICG-derived fluorescence was measured with an in vivo imaging system. White dot lines represent region of interest for the quantification of images with statistical analysis (1: Area = 956, Mean = 0, 2: Area = 1937, Mean = 227, 3: Area = 1936, Mean = 226, 4: Area = 1182, Mean = 178, 5: Area = 956, Mean = 136, 6: Area = 1052, Mean = 102, 7: Area = 853, Mean = 0, 8: Area = 855, Mean = 39, 9: Area = 4856, Mean = 5, 10: Area = 451, Mean = 0, and 11: Area = 1205, Mean = 1).

Next, we evaluated the ability of NPs to accumulate in tumors by the EPR effect. It has been reported that NP particle size significantly affects the EPR effect^25,26^. When the particle size is large, they cannot sufficiently penetrate into tumor tissue and they accumulate in the liver, potentially causing hepatotoxicity. However, when the particle size is too small, they are eliminated from the body more rapidly by renal clearance and cannot sufficiently infiltrate the tumor tissue. Near-infrared fluorescent dye ICG was used to identify the tumor location in CT26 tumor-bearing mice following intravenous injection. Strong ICG-mediated fluorescence was observed using an in vivo fluorescent bioimaging system (Fig. 3). In fact, NPs encapsulating ICG molecules were selectively accumulated within CT26 tumors in a time-dependent manner. After 12 h, CT26 tumors and vital organs (lung, heart, liver, kidneys, and spleen) were collected from the mice. The highest fluorescence intensity was observed in CT26 tumors compared to the other organs. A minor amount of fluorescence was observed in the kidneys. These results indicate that NPs have excellent tumor-targeting ability via the EPR effect and suggest that the major elimination pathway for NPs is renal excretion. Considering this result (Fig. 3) and that the elimination t_1/2_ of KS-58 in blood is approximately 30 min after i.v. administration^14^, t_1/2_ of KS-58 NPs in blood may be prolonged in compared with that of KS-58. Indeed, it is well known that t_1/2_ of nanomedicines were often found to be better than that of pristine drugs without nanoformulations^27^.

### In vivo antitumor efficacy of NPs in a CT26 syngeneic tumor model

**Figure 4:**
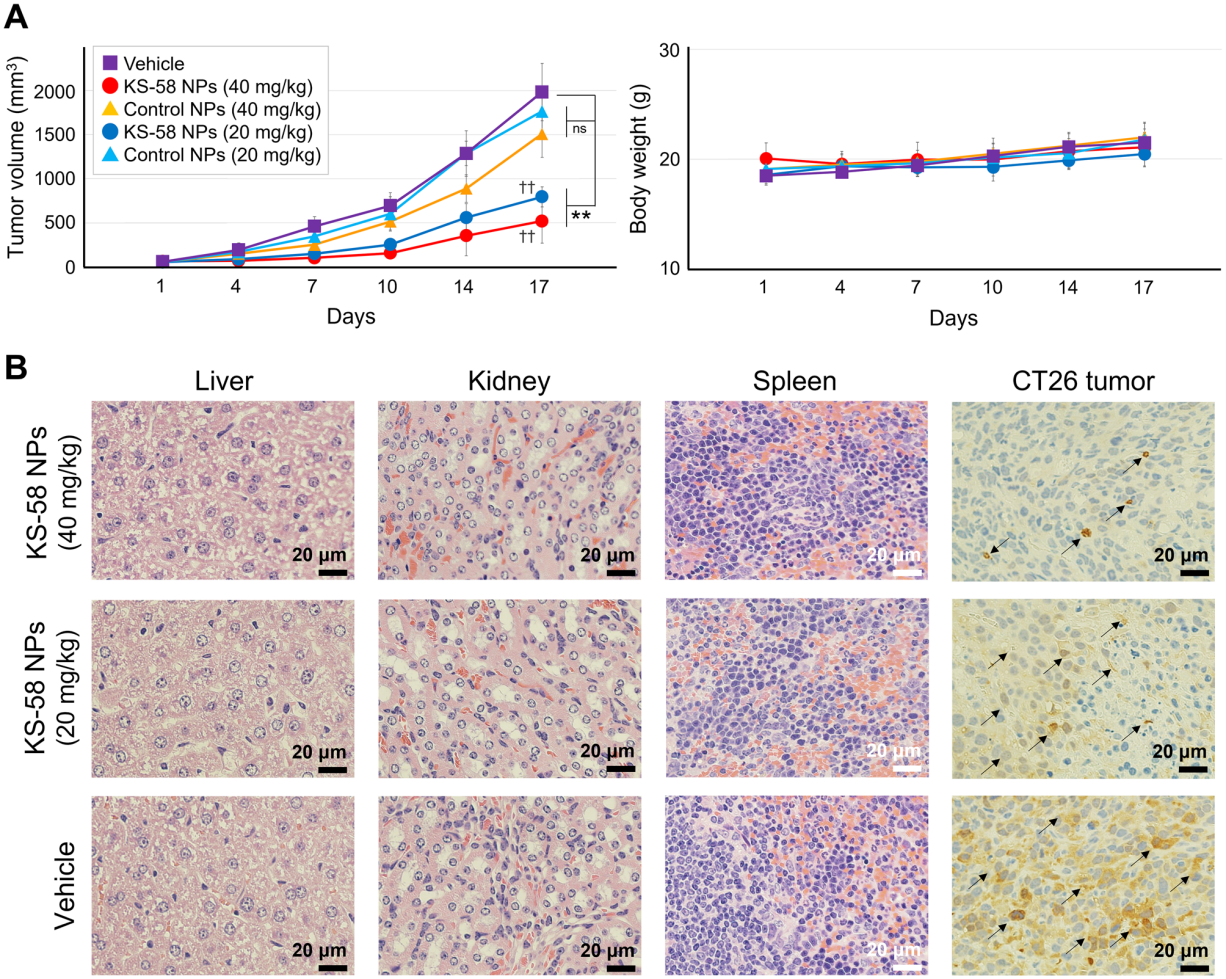
Antitumor efficacy of KS-58 NPs in mice bearing CT26 subcutaneous allografts. (**A**) Changes in tumor volume and body weight (n = 4 mice per group, expressed as mean ± SD, **p < 0.001 vs. Vehicle control by Dunnett’s test, ^††^*p* < 0.001 vs. Control NPs by a Student’s t-test). (**B**) Immunohistochemical (IHC) staining of tissue sections. Liver, kidney, spleen, and CT26 tumors were collected from the mice were fixed in 10% neutral-buffered formalin, embedded in paraffin, and sectioned. Livers, kidneys, and spleens were stained with hematoxylin and eosin. CT26 tumors were stained with an anti-pERK antibody. Black arrows represent color development regarding to the pEPK expression.

The anti-cancer effect of KS-58 NPs on subcutaneously implanted CT26 cell-derived tumors was evaluated (Fig. 4). The tumor size of vehicle group increased progressively following transplantation, but the tumor growth rate was significantly reduced by weekly intravenous administration of KS-58 NPs (KS-58 concentration = 20 and 40 mg/kg) (Fig. 4A and Table 1). Control NPs without KS-58 exhibited a slight trend toward slower tumor growth, but there was no statistically significant difference compared with the vehicle group (Fig. 4A and Table 1). No significant weight gain or loss of mice compared to the vehicle group was observed in either group during the study period. The mean tumor volume of KS-58 NPs (20 mg/kg) and KS-58 NPs (40 mg/kg) groups was 40 ± 6% and 26 ± 12%, respectively, whereas that of vehicle group was set to 100% (Table 1). The mean tumor volumes of the KS-58 NPs (20 mg/kg) and KS-58 NPs (40 mg/kg) groups were 45 ± 6% and 34 ± 16%, respectively, whereas that of the control NPs group were set to 100%, respectively (Table 1). In a previous report^14^, KS-58 (40 mg/kg, every 2 days administration) suppressed CT26 tumors growth to 35% relative to the vehicle group. In the present study, KS-58 NPs (40 mg/kg, once a week administration) suppressed CT26 tumor growth to 26% relative to the vehicle group.

**Table 1:** CT26 tumor growth rate in vivo.

Vehicle	Control NPs (20 mg/kg)	Control NPs (40 mg/kg)	KS-58 NPs (20 mg/kg)	KS-58 NPs (40 mg/kg)
100 ± 16%	89 ± 12%	76 ± 13%	40 ± 6%***	26 ± 12%***
–	100 ± 14%	–	45 ± 6%^†††^	–
–	–	100 ± 17%	–	34 ± 16%^†††^

Growth rate (%) was calculated based on the tumor volume of the vehicle group at day 17 as 100% and expressed as the mean ± SD (n = 4, ***p < 0.001 vs. Vehicle group by Dunnett’s test, ^†††^*p* < 0.001 vs. Control NPs group by a Student’s t-test).

We found that KS-58 NPs inhibited K-Ras(G12D) activity by reducing ERK phosphorylation (pERK), which is a Ras-mediated downstream signaling event, as determined by immunohistochemical (IHC) staining of CT26 tumor tissue sections (Fig. 4B). KS-58 NPs markedly decreased the expression (color development) of pERK in a dose-dependent manner. These data suggest that tumor growth suppression occurs through the inhibition of K-Ras(G12D)-mediated signaling. Notably, IHC staining of vital organ tissue sections (liver, kidneys, and spleen) indicated that they were healthy and no side effects were observed following weekly administration of KS-58 NPs (Fig. 4B). In fact, hematoxylin & eosin (H&E) staining of the vital organs of the mice exhibited no obvious signal of tissue damage or inflammation lesion in all treatment groups, further confirming the excellent biocompatibility of as-designed KS-58 NPs.

### In vivo antitumor efficacy of NPs for PANC-1 orthotopic tumor model

**Figure 5:**
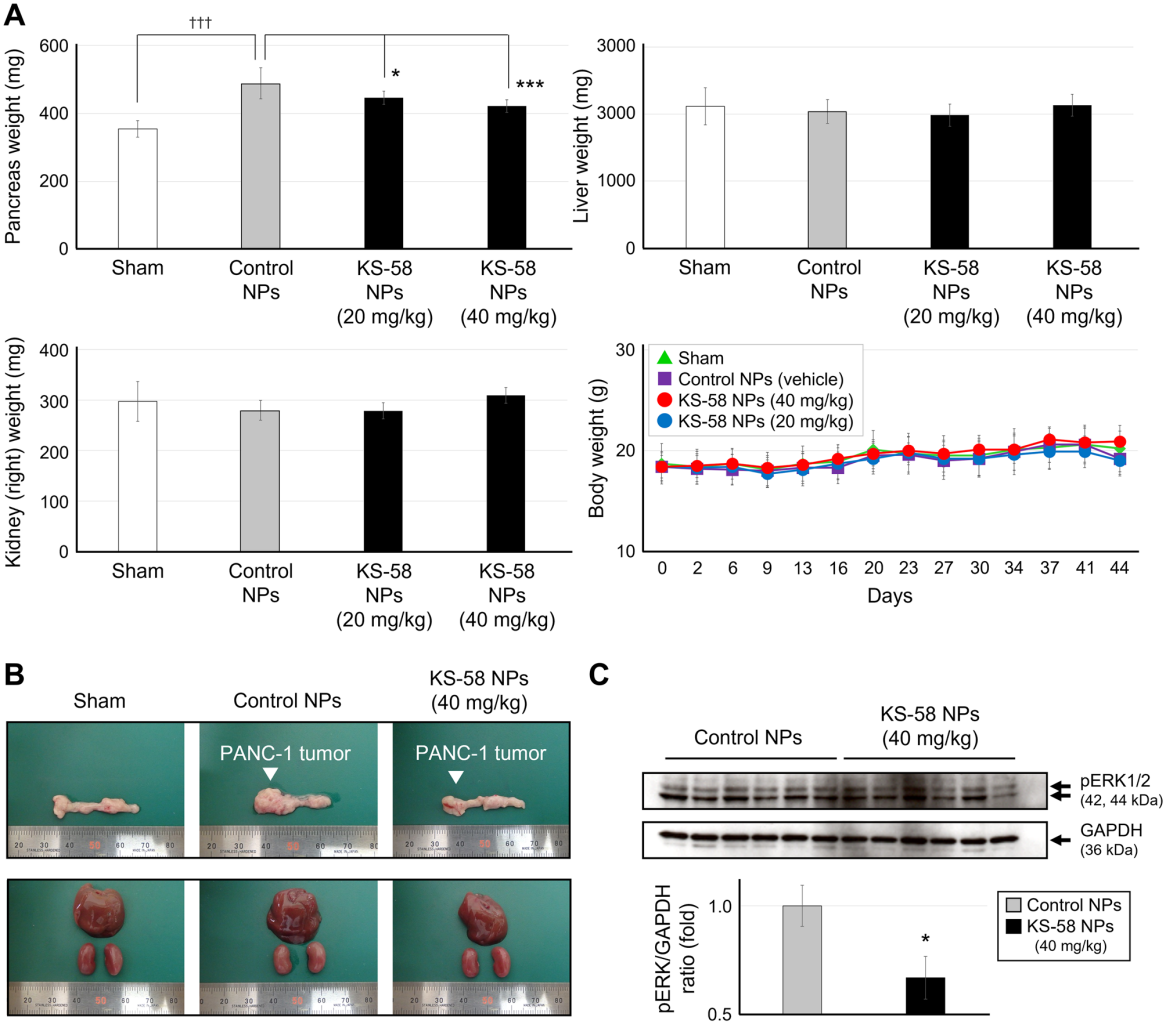
Antitumor efficacy of KS-58 NPs in mice bearing PANC-1 orthotropic xenografts. (**A**) Weight of pancreas, liver, and kidneys (right) collected from the mouse groups on day 44 (n = 8, *p < 0.05, ***p < 0.001 vs. Control NPs group by Dunnett’s test, ^†††^*p* < 0.001 vs. the Sham group by a Student’s t-test) and changes in body weight (n = 8 mice per group, expressed as mean ± SD). (**B**) Representative appearance of each organ (left: pancreas, right: liver and kidneys) from mice in the orthotopic xenografts experiments. (**C**) PANC-1 tumors collected from control NPs group and KS-58 NPs group were homogenized and phosphorylated ERK (pERK) and GAPDH were quantified by Western blotting. Expression levels of pERK were normalized by GAPDH and presented as the fold change relative to the control NPs group (n = 6 tumors per group, ± SEM, *p < 0.05 by a Student’s t-test).

Finally, the antitumor activity of KS-58 NPs in PANC-1 cell-derived tumors implanted into the pancreas of mice was evaluated (Fig. 5). As shown in Fig. 4, various nanomedicines have shown pharmacological efficacy in subcutaneous tumor-bearing mouse models; however, there are few reports in pancreatic cancer models^28,29^. One of the reasons is that angiogenesis occurs in subcutaneously implanted tumors, which facilitates the EPR effect; however, tumors formed in the pancreas are covered with stroma and have few blood vessels making it difficult to demonstrate an EPR effect^28,29^. KS-58 NPs showed a clear pharmacological effect not only in subcutaneous tumor-bearing mice (Fig. 4) but also in pancreatic cancer mouse models (Fig. 5). A weekly intravenous dose of KS-58 NPs (20 and 40 mg/kg) resulted in significant tumor growth suppression in xenografts without adverse side effects, such as body weight loss or organ (liver and kidney) swelling (Fig. 5A,B). To confirm that antitumor activity of KS-58 NPs was caused by K-Ras(G12D) inhibition, western blotting was conducted. Six tumors were randomly selected from each of the control NPs group and KS-58 NPs (40 mg/mL) group. A total of 12 tumors were homogenized and subjected to Western blotting to quantify pERK and GAPDH (Fig. 5C). As a result, a significant reduction of pERK/GAPDH ratio of KS-58 NPs group in compared with that of control NPs group. Antitumor efficacy of KS-58 NPs may have occurred because the KS-58 NPs particle size is around 10 nm, which is small enough to infiltrate readily into pancreatic tumor tissue, but of sufficient size to delay renal excretion. It has been reported that large NP particle size decreases penetration ability into tumor tissue^25,26^. However, when the particle size is too small, they are eliminated from the body more rapidly by renal clearance. Choi et al. reported that quantum dots (particle size < 5.5 nm) are early eliminated by renal clearance^30^. Poon et al. reported that nanoparticle formulations with a particle size of 5.5 nm or larger delay elimination from the kidneys^31^. The mean tumor volumes of the KS-58 NPs (20 mg/kg) and KS-58 NPs (40 mg/kg) groups were 68 ± 15% and 50 ± 14%, respectively, compared with that of the Sham and control NPs groups, which were set at 0% and 100%, respectively (Table 2). In a previous report^13^, KS-58 (40 mg/kg administered every 2 days) suppressed PANC-1 tumors growth to 65% relative to the vehicle group. In the present study, KS-58 NPs (40 mg/kg, once a week administration) suppressed PANC-1 tumors growth to 50% relative to the control NPs group.

**Table 2:** PANC-1 tumor growth rate in vivo.

Sham	Control NPs (40 mg/kg)	KS-58 NPs (20 mg/kg)	KS-58 NPs (40 mg/kg)
0 ± 18%	100 ± 34%^†††^	68 ± 15%*	50 ± 14%***

Growth rate (%) was calculated based on the pancreas weight of the Sham group as 0% and the Control NPs group as 100% and expressed as the mean ± SD (n = 8, *p < 0.05, ***p < 0.001 vs. Control NPs group by Dunnett’s test, ^†††^*p* < 0.001 vs. Sham group by a Student’s t-test).

Figures 4 and 5 indicate that the nanoformulation enabled not only a reduction in the frequency of administration but also an improvement in the efficacy of KS-58. Smaller-sized nanoparticles are reportedly less immunogenic^32,33^. KS-58 NPs have been administered multiple times to both wild-type (Fig. 4) and immunodeficient mice (Fig. 5) and have shown pharmacological efficacies in both types of mice. Although the immunogenicity of KS-58 NPs should be further evaluated in the future, the fact that its anti-cancer effect was not lost suggests that KS-58 NPs are not eliminated by the immune system.

## Conclusion

In summary, we succeeded at circumventing the water solubility and frequent dosing problems associated with KS-58, which have prevented the successful development of KS-58, by producing a nanoformulation using CrEL. KS-58 NPs exhibited significant antitumor activity not only against subcutaneous tumor-bearing mice but also pancreatic cancer mouse xenografts using a once-weekly administration without any notable side effects. The nanoformulation of KS-58 represents a new therapeutic strategy against intractable cancers, such as colorectal and pancreatic cancers that express K-Ras(G12D).

## Supplementary Information

Supplementary Figures.

## Data Availability

All data generated in this study are contained in the manuscript. Raw data are available from the corresponding author upon reasonable request.
