# Supplementary material for: Nanoformulation of the K-Ras(G12D)-inhibitory peptide KS-58 suppresses colorectal and pancreatic cancer-derived tumors
Source: Sci Rep. 2023 Jan 10;13:518. doi: 10.1038/s41598-023-27825-8 (PMC9832047; doi:10.1038/s41598-023-27825-8)

Nanoformulation of the K-Ras(G12D)-inhibitory peptide KS-58 suppresses colorectal and pancreatic cancer-derived tumors

Kotaro Sakamoto*[1], Yun Qi[2], and Eijiro Miyako*[2]

[1]Research & Development Depertment, Ichimaru Pharcos Company Limited, 318-1 Asagi, Motosu, 501-0475 Gifu, Japan

[2]Graduate School of Advanced Science and Technology, Japan Advanced Institute of Science and Technology, 1-1 Asahidai, Nomi, 923-1292 Ishikawa, Japan

*Corresponding authors:
Kotaro Sakamoto: sakamoto-kotaro@ichimaru.co.jp
Eijiro Miyako: e-miyako@jaist.ac.jp

Supplementary Figure S1. Stability of KS-58 NPs.

A Cell growth suppression activity of KS-58 NPs immediately after preparation, after storage at 4°C for one month, and after storage at 25°C for one month. The relative PANC-1 cell proliferation of each group is shown as % values compared with the control, which was set at 100% (n = 4, ± SEM, $^{**}p < 0.01$, n.s means no statistical significance by Dunnett's test). **B** Appearance of KS-58 NPs after storage at 4°C for one month, and after storage at 25°C for one month.

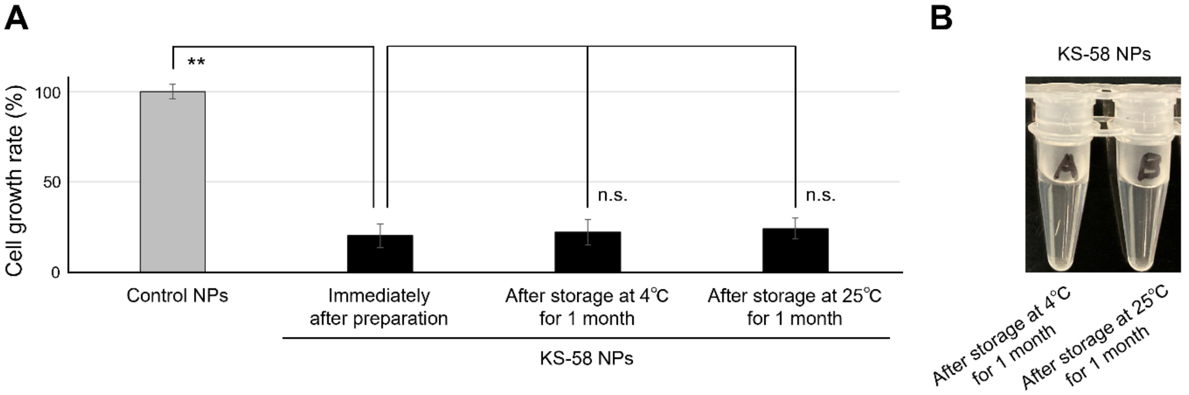

Supplementary Figure S2. Size distribution of KS-58 NPs and control NPs.

The hydrodynamic diameters of the KS-58 NPs and control NPs were approximately 12.95 nm and 10.02 nm, respectively.

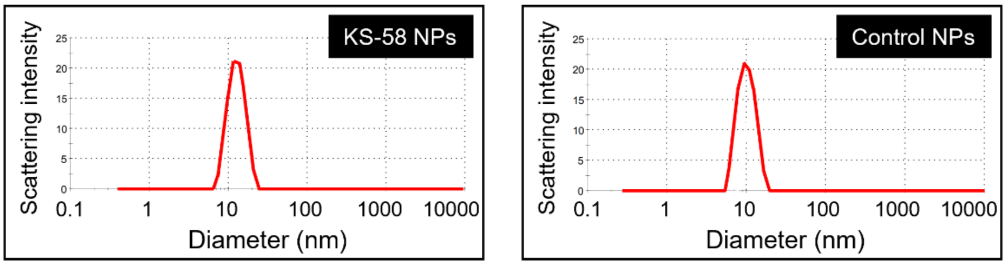

Supplementary Figure S3. Full length blots of Figure 5C.

pERK

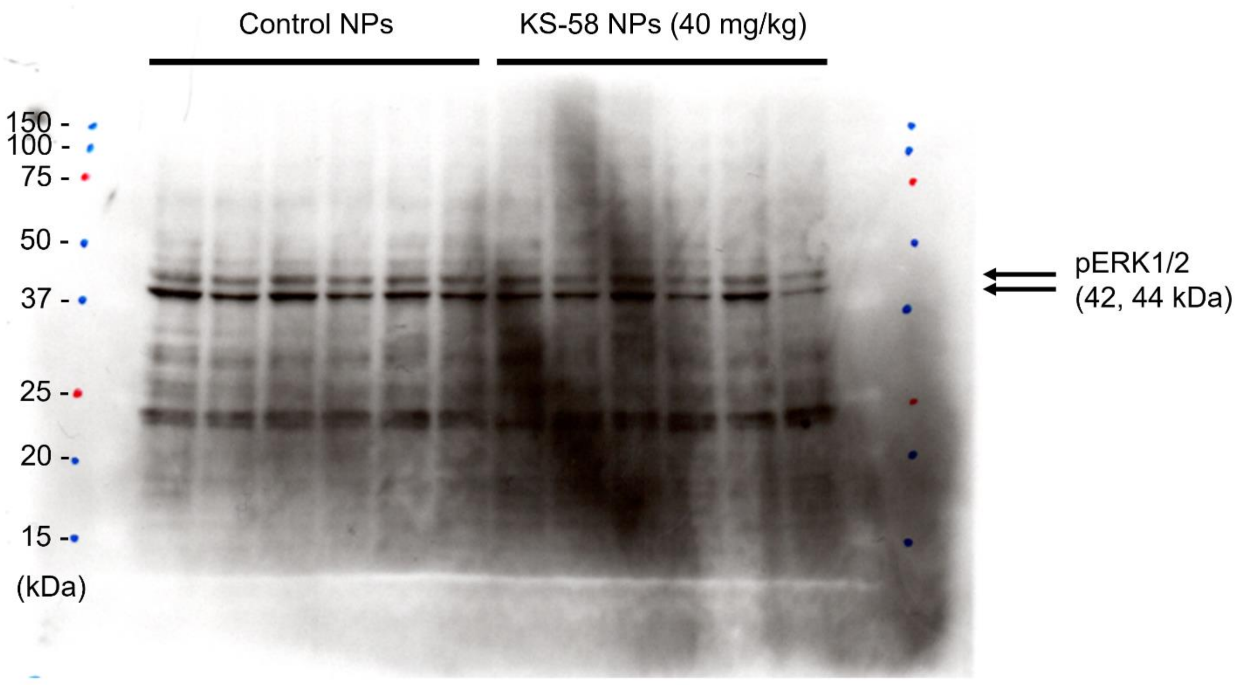

GAPDH

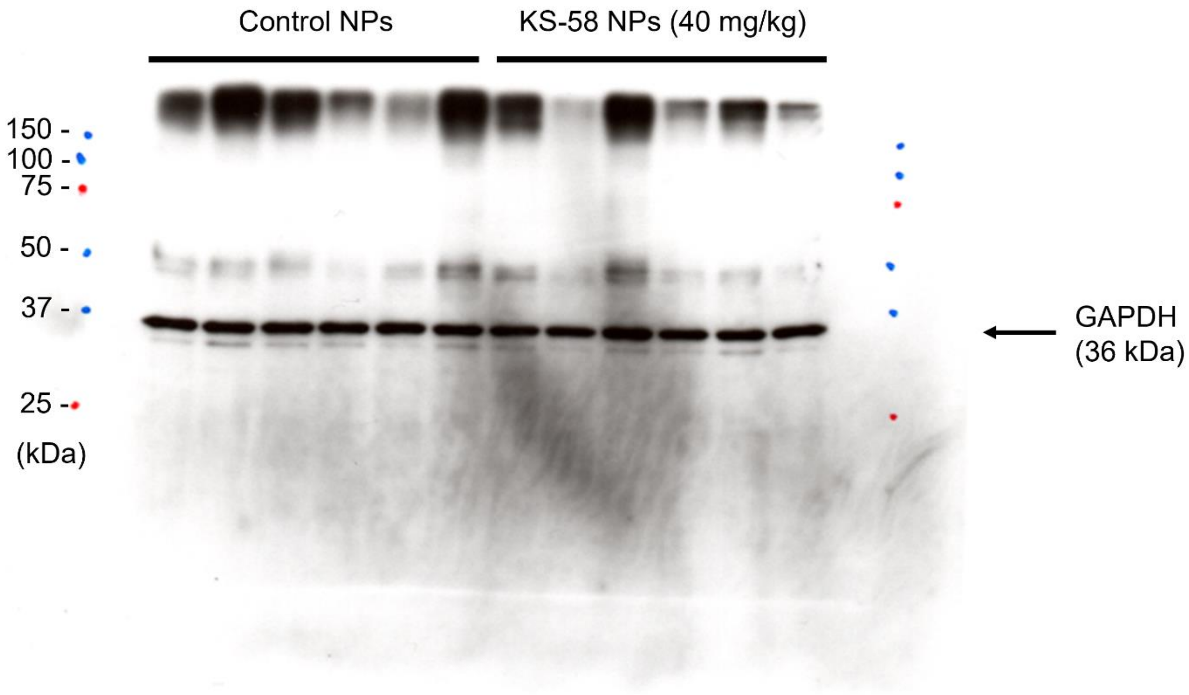

Supplementary Figure S4. Appearance of the KS-58 NPs 24 h after dilution.

KS-58 NPs were prepared as 2-fold dilution series by 5% glucose, saline, D-PBS, or purified water.

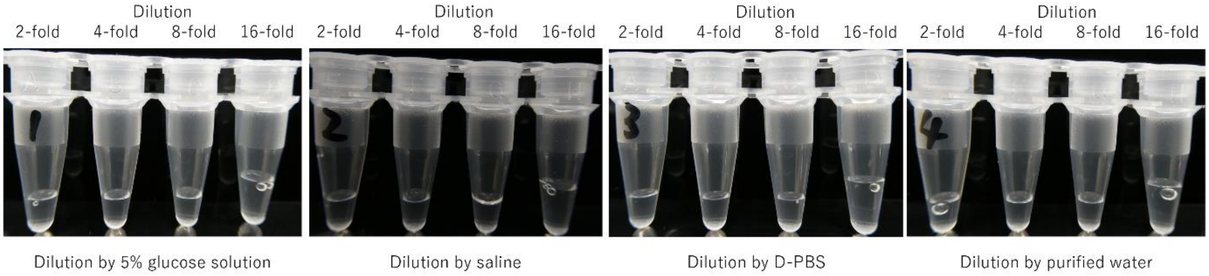

Supplement: Supplementary file 1 — Supplementary Figures. [file 41598_2023_27825_MOESM1_ESM.pdf]
